# Effects of nateglinide and rosiglitazone on pancreatic alpha- and beta-cells, GLP-1 secretion and inflammatory markers in patients with type 2 diabetes: randomized crossover clinical study

**DOI:** 10.1186/s13098-015-0120-6

**Published:** 2016-01-04

**Authors:** Glauce Cordeiro Ulhôa Tostes, Maria Rosário Cunha, Rosa Tsumeshiro Fukui, Márcia Regina Silva Correia, Dalva Marreiro Rocha, Rosa Ferreira dos Santos, Maria Elizabeth Rossi da Silva

**Affiliations:** Laboratório de Carboidratos e Radioimunoensaios LIM-18 do Hospital das Clínicas da Faculdade de Medicina da, Universidade de São Paulo, São Paulo, Brazil

**Keywords:** Nateglinide, Rosiglitazone, GLP-1, Haemostatic factors, Inflammatory markers, Type 2 diabetes

## Abstract

**Background:**

To compare the effects of nateglinide and rosiglitazone on inflammatory markers, GLP-1 levels and metabolic profile in patients with type 2 diabetes (DM2).

**Methods:**

A prospective study was performed in 20 patients with DM2, mean age 51.82 ± 8.05 years, previously treated with dietary intervention. Participants were randomized into rosiglitazone (4–8 mg/day) or nateglinide (120 mg 3 times a day) therapy. After 4 months, the patients were crossed-over with 8 weeks washout period to the alternative treatment for an additional 4-month period on similar dosage schedule. The following variables were assessed before and after 4 months of each treatment period: (1) a test with a standardized 500 calories meal for 5 h including frequent measurements of glucose, insulin, glucagon, proinsulin, GLP-1, free fat acids (FFA), and triglycerides levels was obtained. The lipid profile and HbA1 levels were measured at fasting. (2) Haemostatic and inflammatory markers: platelet aggregation, fibrinogen, PAI-1 activity, C reactive protein (CRP), IL-6, TNF-α, leptin, sICAM and TGFβ levels.

**Results:**

Both therapy decreased blood glucose levels under the postprandial curve but neither affected glucagon and GLP-1 levels. Nateglinide was associated with higher insulin and pro-insulin secretion, but similar pro-insulin/insulin ratio when compared with rosiglitazone. Only rosiglitazone decreased Homa β, PAI-1 activity, CRP, fibrinogen, TGFβ, FFA and triglyceride levels.

**Conclusions:**

Nateglinide and rosiglitazone were effective in improving glucose and lipid profile and β cell function, but rosiglitazone afforded a better anti-inflammatory effect. No drug restored alpha cell sensitivity or changed GLP-1 levels. Maintenance of haemostatic factors, inflammatory factors and glucagon levels can be related to the continuously worsening of cardiovascular function and glucose control observed in DM2.

## Background

Cardiovascular disease is the leading cause of mortality in patients with type 2 diabetes mellitus. The rigorous control of glycemia can delay but not prevent vascular complications, which are probably related to many other poorly controlled atherogenic factors, such as obesity, hypertension, hyperlipidemia, insulin resistance, oxidative stress, accelerated aging, disturbances in coagulation and fibrinolysis [1].

Although metformin has been confirmed as the first line option to treat diabetes, troublesome gastrointestinal intolerance sometimes precludes its use [2].

Rosiglitazone and nateglinide are drugs that differ in their primary mechanism of action and have been previously considered in type 2 diabetes treatment.

The thiazolidinedione rosiglitazone, a peroxisome proliferator activated receptor gamma (PPARγ) agonist, is an insulin sensitizing agent that improves glycaemic control and a variety of other metabolic disturbances in patients with type 2 diabetes. However, besides weight gain, it promoted fluid retention and heart failure [3–5]. The increased risk of acute myocardial infarction and a trend towards increased mortality with the drug brought concern about the safety of rosiglitazone, prompting its withdrawn of the market.

Another alternative option to therapy can be the insulin secretagogue nateglinide [6] that is a derivative of phenylalanine and structurally distinct of sulphonylureas, which also had raised concern over their potential adverse effects in the event of ischemic heart disease. Nateglinide’s interaction with sulpnhonylurea receptor (SUR1), a subunit of the ATP sensitive potassion channel (K_ATP_) on plasma membrane is fleeting, favoring a rapid onset and short duration of insulin secretion, providing post-prandial glucose control with less hypoglycemia and weight gain [6, 7]. The promotion of glucagon like peptide 1-(GLP-1) release from intestinal L cells may be another important mechanism by which nateglinide restores early-phase insulin secretion and regulates postprandial glucose metabolism [8]. However, this effect could be due to improvement in glucose levels and deserves confirmation.

Furthermore, nateglinide has little binding to the vascular muscle and cardiac SUR2 receptors [9] suggesting that it could be a good and safe option to new diagnosed type 2 diabetes. However there are few and contradictory reports of nateglinide effects on cardiovascular function [10–14] and inflammatory markers, many of them comparing nateglinide to placebo, difficulting to separate the impact of improvement of glucose control on the obtained results. Also, the influence of nateglinide on glucagon secretion is poorly known.

To the best knowledge of nateglinide effects we conducted this study comparing the effects of nateglinide with rosiglitazone on glucose and lipid profile, but also on several parameters still not well characterized like pancreatic alpha and beta cells response to diet, the incretin hormone GLP-1, inflammatory markers, and haemostatic factors. The purpose of the current study was also to ascertain whether anti-diabetic agents with different primary mechanisms of action targeting the two main pathophysiological defects of type 2 diabetes would have different effects on these parameters and if they were independent of glucose control.

## Methods

### Subjects

A prospective study was performed in 22 patients with type 2 diabetes according to ADA criteria [2], 16 F:6 M, mean age 51.2 ± 8.05 years, IMC = 27.9 kg/m^2^ and diabetes duration of 1.9 ± 2.1 years that were treated with dietary intervention in the previous 2 months.

### Study design

Participants were randomly assigned to receive either nateglinide (nateglinide group) or rosiglitazone (rosiglitazone group). The rosiglitazone dosage was titrated in order to achieve fasting glucose levels lower than 7.0 mmol/L using domiciliary capillary glucose measurements. Nateglinide was administrated in a fixed dose three times a day. After 4 months, the patients were crossed-over after 8-week washout period to the alternative treatment for an additional 4-month period on a similar dosage schedule. Subjects were followed on an outpatient basis every 1–2 weeks for drug and weight-maintaining diet adjustments throughout the study period. The clinical characteristics of the patients are depicted in Table 1. At the time of entry, a complete history, physical examination, and laboratory evaluation including urinalysis, renal, hepatic and thyroid function tests, serum lipid and electrolytes levels and ECG were obtained for all subjects. None of the patients exercised on a regular basis. Exclusion criteria included any severe concomitant illness, uncontrolled hypertension (blood pressure > 190 × 120 mmHg), marked dyslipidemia and use of hypolipemic and anticoagulant medications. No subjects had any acute concurrent illness.

**Table 1:** Clinical and biochemical characteristics of 20 patients with type 2 diabetes at baseline and after 4 months of nateglinide or rosiglitazone therapy

Variables	Baseline		Nateglinide		p	Baseline		Rosiglitazone		p
			After 4 months					After 4 months		
	Median	(25th–75th)	Median	(25th–75th)		Median	(25th–75th)	Median	(25th–75th)	
Body weight (kg)	67.9	(64.1–81.0)	70.0	(64.5–78.0)	0.073	69.0	(63.9–77.0)	68.9	63.6–77.9	0.984
Hba1c (%)	7.45	(7.0–8.3)	6.65	(5.7–7.8)	*0.032*	7.2	(6.8–8.1)	6.6	5.5–7.5	*0.036*
Homa Beta	40.7	(28.8–52.0)	51.3	(28.7–73.0)	0.126	33.6	(26.8–59.0)	51.6	30.9–69.9	*0.005*
Total-c (mg/dL)	223.5	(178.5–235.3)	194.0	(182.0–213.0)	*0.036*	213.0	(184.0–230.0)	211.0	201.0–245.0	0.277
HDL-c (mg/dL)	44.5	(37.8–57–8)	44.5	(34.0–55.0)	0.360	45.0	(40.0–56.0)	48.0	38.0–55.0	0.347
LDL-c (mg/dL)	144.0	(111.0–153.5)	131.5	(109.5–142.0)	0.145	152.0	(106.0–157.0)	142.0	124.0–168.0	0.355
CRP (mg/L)	5.2	(1.87–8.91)	3.79	(1.63–7.64)	0.052	4.16	(2.23–8.37)	3.10	(0.87–6.34)	*0.042*
TGF β (pg/mL)	44.1	(34.2–51.1)	45.3	(32.2–49.4)	0.247	42.7	(37.8–47.9)	38.3	32.0–45.8	*0.001*
Interleukin-6 (pg/mL)	2.54	(1.45–5.66)	2.37	(1.16–3.53)	0.108	2.71	(1.46–4.17)	2.00	1.42–3.89	0.520
leptina (ng/mL)	14.9	(7.6–21.0)	17.9	(10.0–20.8)	0.808	13.1	(6.0–19.3)	16.2	7.0–24.1	0.199
sICAM-1 (ng/mL)	233.8	(179.7–261.5)	214.9	(186.7–263.3)	0.391	223.6	(171.3–241.6)	218.0	173.8–246.0	0.573
TNF α (pg/mL)	1.71	(1.18–2.50)	1.52	(1.08–2.91)	0.235	1.49	(1.23–2.09)	1.43	1.18–2.18	0.760
fibrinogen (mg/dL)	316.0	(276.8–375.0)	336.5	(297.8–357–5)	0.433	337.0	(296–397)	322.0	(262.5–363.8)	*0.024*
PA-1 activity (UI/mL)	27.7	(14.4–43.8)	24.7	(14.7–40.3)	0.55	15.9	(11.6–40.0)	12.7	8.0–19.0	*0.040*
platelet aggregation	90.3	(80.1–96.0)	90.0	(81.0–92.6)	0.601	89.0	(76.4–94.5)	90.9	76.0–95.0	0.643

Wilcoxon matched pairs tests were performed to compare the differences before and after the 4 months of therapy for each group

Italic values indicate statistical significance at p < 0.05

*CRP* reactive protein, *HDL-c* HDL cholesterol, *LDL-c* LDL-cholesterol, *PAI-1* plasminogen activator inhibitor, *sICAM-1* intercellular adhesion molecule-1, *TGFβ* transforming growth factor beta, *TNFα* tumor necrosis factor alfa, *Total-c* total cholesterol

The Medical Ethics Committee of Hospital das Clínicas da Faculdade de Medicina da Universidade de São Paulo approved the study protocol and all subjects gave written informed consent.

### Study protocol

The patients were instructed to follow similar food intake and to abstain from use of tobacco, alcohol, coffee and any physical activity 24-h before the test days. The following procedures were performed before (basal values) and after each 4 months treatment period (nateglinide or rosiglitazone groups): (1) hormonal and metabolic determinations: a test with a standardized 500-kcal mixed breakfast tolerance test (60 % carbohydrate, 20 % fat and 20 % protein) for 5 h including frequent plasma or serum measurements (at times 0, 15, 30, 45, 60, 120, 180, 240 and 300 min) of glucose, insulin, glucagon, proinsulin, GLP-1, free fat acids (FFA), and triglycerides levels was performed after 12 h fasting. The fasting lipid profile and HbA_1_ levels were also measured. (2) Haemostatic and inflammatory markers: fasting blood fibrinogen, plasminogen activator inhibitor (PAI-1) activity, C reactive protein (CRP), Interleukin 6 (IL-6), tumor necrosis factor alfa (TNFα), leptin, intercellular adhesion molecule-1 (sICAM), transforming growth factor beta (TGFβ) levels and platelet aggregation. (3) Cardiovascular evaluation: 24-h blood pressure monitoring.

### Biochemical and hormonal analysis

Glucose levels were determined by the glucose oxidase/peroxidase method (Labtest, São Paulo, Brazil) and glycated hemoglobin (HbA1c) by HPLC (National Glyco Hemoglobin Standardization Program, USA. Triglyceride levels were measured by the lipase/glycerol kinase method (Labtest, São Paulo, Brazil) and total cholesterol (total-C) by the cholesterol oxidase/peroxidase method. HDL-cholesterol (HDL-C) was separated using the phosphotungstic acid/Mg^2+^ method and measured by oxidase/peroxidase method. LDL-cholesterol (LDL-C) was estimated by Friedewald equation. Free fat acids (FFA) were measured utilizing enzymatic colorimetric method (Wako Chemicals USA, INC). A double-antibody radioimmunoassay was used to measurements of. insulin, proinsulin, glucagon (Linco Research, St. Louis, MO, USA) and total GLP-1 (Millipore Corporation. Billerica, MA) Intra-assay and interassay CVs for hormonal analyses were 6.8 and 9.6 % for insulin; 4.4 and 6.5 % for glucagon; 5 and 5.3 % for proinsulin and <5 % for GLP-1. Circulating levels of Il-6, TNF-α, leptina, sICAM, TGF beta and CRP were measured with high sensitivity ELISA kits (R&D Systems). The samples of each patient were analyzed in the same assay. Intra-assay CV were <8 % for Il-6, TNF-alfa and leptin and <4.5 % for sICAM, TGF-β and CRP.

Haemostatic factors were measured using the same assay for each patient. Fibrinogen was determined by the CLAUSS method [15] with Fibriquick Assay, Sigma, USA. The plasminogen activator inhibitor (PAI-1) activitiy was determined by quantitative assays (Chromolize™ PAI-1, Biopool, Umea, Sweden); The intra-assay CVs were 8 and 3.7 %, respectively. Platelet aggregation was also determined [16].

Systolic and diastolic blood pressure (BP) values were assessed by 24-h ambulatory BP monitoring in all patients every 20 min from 8.00 a.m. to midnight, and every 30 min from midnight to 8.00 a.m. in the following day.

All analyses were done in duplicate.

### Statistical analysis

Numerical data were reported as mean and standard deviation or median and percentile. Differences (95 % CI) between treatment groups were initially tested for treatment-time interaction [17] and then compared by Mann–Whitney tests. Baseline and post-treatment differences between the nateglinide and rosiglitazone groups were compared by Mann–Whitney tests. Wilcoxon matched pairs tests were performed to compare the differences in clinical and biochemical measurements for each group before and after the four months of therapy. The responses to the standardized 500-kcal mixed breakfast tolerance test were analyzed by the area under the curve using the trapezoidal rule and by Wilcoxon test for every time for the 5 h duration. Graphical representations are as mean and standard error. Statistical analysis was performed using Prisma software (version 10.1, SPSS Inc., Chicago). In all cases a p < 0.05 was considered statistically significant.

## Results and discussion

### Results

No treatment period interaction effect was demonstrated; hence the values from each treatment periods were analyzed together. Two patients that started with rosiglitazone did not finished the nateglinide period due to a worsening in diabetes control. The final analysis comprised 20 patients on rosiglitazone and 20 patients on nateglinide.

#### Anthropometric, biochemical, hormonal and haemostatic factors measurements

There were no significant differences (p > 0.05) in baseline clinical and biochemical measurements of those patients randomized to nateglinide or rosiglitazone (Table 1).

HbA1 levels fell (nateglinide-p = 0.032; rosiglitazone-p = 0.036) equally in both treatment groups (p = 0.91). Proportions of patients achieving an endpoint HbA1c of <7 % were similar for nateglinide (60 %) and rosiglitazone (75 %) groups p = 0.5.

The total and incremental areas under the curve (AUC) during the standardized diet tolerance test were analyzed. Both treatments caused a similar decrease in total and incremental prandial glucose AUCs (Table 2).

**Table 2:** Test with a standardized 500 calories mixed breakfast for 5 h: metabolic profile

Areas under the curve	Baseline		Nateglinide		p	Baseline		Rosiglitazone		p
			After 4 months					After 4 months		
	Median	(25th–75th)	Median	(25th–75th)		Median	(25th–75th)	Median	(25th–75th)	
Glucose (mg/dL × min)										
Total	52,455	(4668–60,140)	40,245	(35,468–48,125)	*0.001*	47,340	(41,770–57,240)	43,670	37,820–51,550	*0.005*
Incremental	11,955	(9251–15,523)	9241	(8004–12,805)	*0.005*	12,620	(8216–16,070)	11,070	(7595–12,330)	*0.035*
Insulin (U/mL × min)										
Total	6454	(5561–9922)	9220	(5807–16,340)	*0.008*	6992	(5023–8584)	6120	4213–7590	0.099
Incremental	4107	(3277–6917)	6442	(4255–12,038)	*0.006*	4051	(2566–5324)	3208	2658–4756	*0.049*
Proinsulin (pM × min)										
Total	15,460	(9841–27,613)	16,020	(10,705–30,395)	0.135	12,470	(10,230–24,500)	11,730	7823–18,830	*0.033*
Incremental	8100	(5601–12,305)	9915	(6203–18,713)	*0.03*	6668	(5505–9680)	6263	4706–9455	0.107
Glucagon (pg/mL × min)										
Total	23,535	(21,070–27,958)	22,160	(19,023–27,795)	0.455	21,460	(16,470–27,140)	24,060	19,500–25,740	0.520
Incremental	3565	(2739–5480)	3094	(2119–5031)	0.351	3647	(2039–4770)	3268	2344–4302	0.748
GLP1 (pM × min)										
Total	46,995	(36,463–53,685)	48,660	(36,773–56,098)	0.179	42,890	(32,640–56,060)	42,390	37,710–47,390	0.872
Incremental	4140	(3125–5464)	4454	(2680–6693)	1.000	3840	(2065–6469)	4171	2491–5602	0.687
Triglicerides (mg/dL × min)										
Total	43,500	(30,578–64,283)	46,375	(35,430–61,880)	0.737	42,060	(32,320–54,510)	40,350	30,480–59,240	0.717
Incremental	9353	(5474–15,643)	11,120	(9132–16,548)	0.218	10,080	(6660–13,170)	9106	3945–10,580	*0.044*
Free fat acids (mEq/L ×min)										
Total	85.4	(75.6–113.6)	98.0	(81.5–119.1)	0.263	105.9	(79.1–113.3)	70.5	52.7–80.0	*0.007*
Glucose/insulin ratio										
Total	6.55	(5.27–10.01)	4.37	(2.86–6.62)	*0.001*	7.32	(5.93–10.61)	6.87	5.62–10.39	0.629
Incremental	2.57	(1.72–3.98)	1.69	(0.77–2.31)	*0.001*	2.70	(2.26–5.07)	2.78	1.48–4.45	0.398
Proinsulin/insulin ratio										
Total	2.47	(1.37–3.11)	1.65	(1.37–2.17)	*0.028*	2.2	(1.64–3.2)	2.06	1.36–2.44	0.107
Incremental	1.93	(1.21–2.43)	1.51	(1.20–1.82)	*0.044*	1.84	(1.44–2.40)	1.62	1.37–2.42	0.469
Insulinogenic index										
	48.6	(29.1–80.6)	205.7	(80.0–541.1)	*0.002*	41.8	(26.3–101.9)	65.1	47.8–91.7	0.355

Wilcoxon matched pairs tests were performed to compare the differences for each group before and after the 4 months of therapy

Italic values indicate statistical significance at p < 0.05

*GLP-1* glucagon like peptide-1

**Fig. 1:**
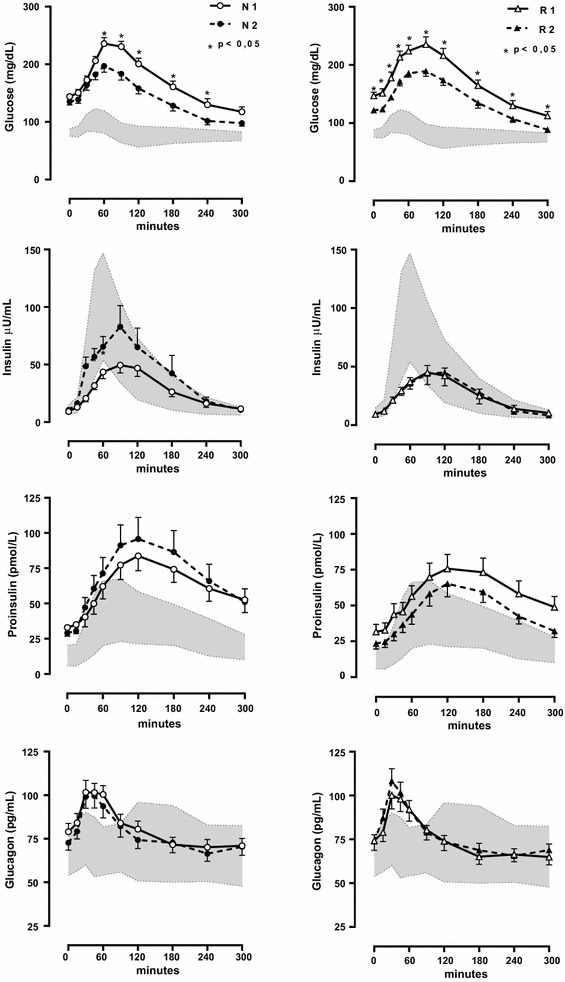
Test with a standardized 500 calories mixed breakfast for 5 h: blood glucose, insulin, proinsulin and glucagon levels. *N1* and *N2* before and after 4 months of nateglinide therapy. *R1* and *R2* before and after 4 months of rosiglitazone therapy. *Hatched area* data of normal controls. Data were analyzed by the area under the *curve* using the trapezoidal rule and by Wilcoxon test for every time for the 5 h duration (p < 0.05)

However, there were significant between-treatment difference for the following parameters: rosiglitazone decreased glucose levels significantly at all times of the curve, including fasting, whereas nateglinide decreased mainly the post prandial glucose levels (Fig. 1).

The initial insulin response to a meal was anticipated and augmented with nateglinide treatment (Fig. 1). Nateglinide also increased the prandial insulin (total and incremental AUC) and pro-insulin (incremental AUC) responses and the insulinogenic index, whereas decreased both glucose/insulin and proinsulin/insulin ratios (total and incremental) Table 2.

On the contrary, rosiglitazone decreased insulin (incremental) and proinsulin (total) AUCs and increased HOMA beta. Comparing both groups, rosiglitazone treatment caused lower total (p = 0.012) and incremental (p = 0.005) insulin AUC, incremental (p = 0.023) pro-insulin AUC and insulinogenic index (p = 0.002), but higher incremental glucose/insulin ratio AUC (p = 0.042) Table 2.

**Fig. 2:**
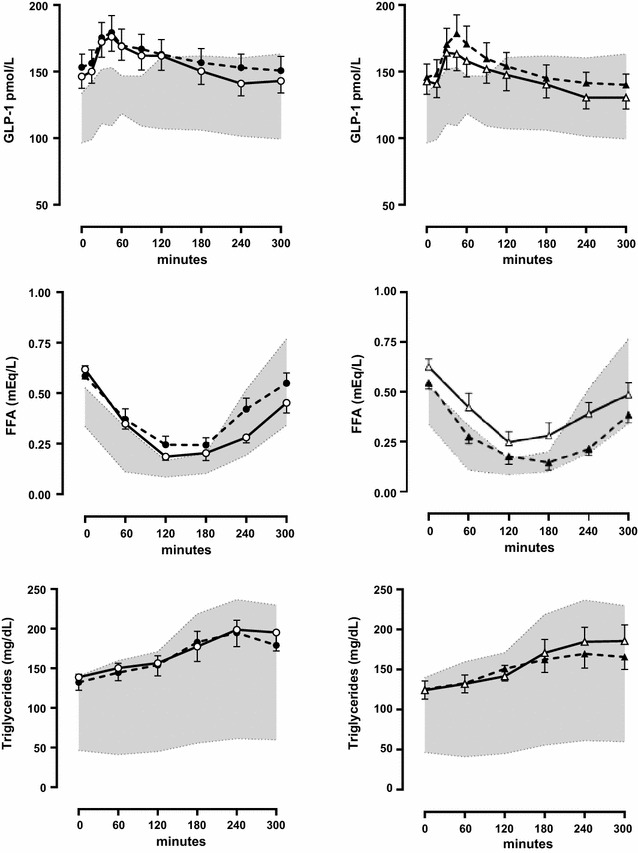
Test with a standardized 500 calories mixed breakfast for 5 h: blood glucagon like peptide-1(GLP-1), free fat acids (FFA) and triglyceride levels. *N1* and *N2* before and after 4 months of nateglinide therapy. *R1* and *R2* before and after 4 months of rosiglitazone therapy. Hatched area: data of normal controls. Data were analyzed by the area under the *curve* using the trapezoidal rule and by Wilcoxon test for every time for the 5 h duration (p < 0.05)

Considering lipids, Rosiglitazone decrease prandial FFA (total AUC) and triglyceride (incremental AUC) (Table 2; Fig. 2), whereas nateglinide decreased fasting LDL-cholesterol levels (Table 1).

Only rosiglitazone decreased PAI-1 activity, fibrinogen, PCR and TGF-β levels (Table 1).

There was no significant effect of either of the therapies on glucagon or GLP-1 AUCs (Table 2; Figs. 1, 2) or on HOMA IR, fasting IL-6, leptin, s-ICAM, TNF alpha and HDL cholesterol levels (Table 1).

Weight and waist/hip ratio did not change after 4 months of both treatments.

#### Twenty-four hour ambulatory blood pressure monitoring

Neither therapy changed 24-h systolic and diastolic blood pressure measurements.

## Discussions

This clinical, prospective, randomized, crossover study compared the effects of two different classes of drugs (rosiglitazone, an insulin sensitizer, and nateglinide, an insulin secretagogue) in type 2 diabetes patient. We evaluated the same patient under basal conditions, i.e., while hyperglycemic without medications, and again after 4 months of treatment with nateglinide or rosiglitazone. The major aims of this strategy was to minimize the influence of metabolic control on the specific drug effect. Despite similar improvements on glucose control, changes on lipid profile, insulin response and inflammatory markers were different with therapies.

Both treatment groups achieved similar and significant mean decreases from baseline in plasma glucose and fasting HbA1c levels. Proportions of patients achieving an endpoint HbA1c of <7 % were similar for nateglinide and rosiglitazone groups. Initial insulin response to the 5 h standardized 500 calories breakfast was augmented only with nateglinide treatment. The higher insulin and proinsulin levels, observed during nateglinide therapy, are in line with reports of its stimulatory effects on beta cells function, favoring a quick onset and short duration of insulin secretion, contrasting with the rosiglitazone’s sparing effects on beta cells function [3, 6, 7]. As expected, nateglinide anticipated the insulin peak response, improving mainly post-prandial glucose levels whereas rosiglitazone decreased glucose levels significantly at all times of the curve (Fig. 1).

Similar results were seen with proinsulin levels during the meal test: increased with nateglinide and decreased with rosiglitazone.

So, both treatment affected beta cell function. Despite increasing insulin secretion, the pro-insulin/insulin area under the curve ratio during the 5 h of the breakfast decreased during nateglinide treatment, suggesting that it improved previous secretory dysfunction of beta cells, contrasting with the worsening in beta cells function usually reported to sulphonylureas [18]. The increase in insulin secretion could not be accounted for by changes in body weight—it was unaffected in both groups, probably because of the short duration of post prandial insulin secretion during nateglinide therapy, the frequent ambulatory visits and nutritional counseling of the patients. There was no complains of hypoglycemia with both treatments.

Rosiglitazone also improved beta cells function, attested by the increase in HOMA-B value. Despite causing lower prandial insulin response, rosiglitazone kept total and incremental glucose AUC at similar levels when compared with nateglinide.

Overall changes on plasma lipids levels were different with therapies. Only rosiglitazone decreased post-prandial FFA and of triglycerides levels, both of which probably accounted to the observed amelioration of beta cell function (HOMA-B) and of insulin resistance, reflecting on the glucose-lowering action of rosiglitazone.

By the other side, nateglinide decreased fasting cholesterol levels, that were lower than with rosiglitazone treatment (p = 0.04).

Small changes in lipid metabolism were expected, and previous reports have also been light [3–6]. The near normal triglyceride and cholesterol levels of our patients prior both therapies was probably a factor influencing these modest results. However, neither the increase in insulin production nor the increase in insulin sensitivity were able to decrease fasting or post-prandial glucagon levels, confirming that both the inherent effects of each drugs, allied to the decrease in gluco and lipotoxicity were insufficient to restore alfa cells sensitivity to glucose and insulin levels, favoring the progression of diabetes.

Besides improvements on plasma FFA levels and HOMA beta, rosiglitazone treatment decreased CRP, TGF beta and fibrinogen levels and PAI-1 activity. These data suggest drug specific effects, not dependent on amelioration of the metabolic milieu.

Inflammatory markers and other haemostatic factor (IL-6, TNF alpha, leptin, s-ICAM, platelet aggregation) were unaffected by both treatment, despite the great improvement in glucose profile, Such persistence in inflammation is probably implicated in the poor reduction in cardiovascular disease despite glucose control in type 2 diabetes patients [19].

No changes were observed in GLP-1 levels during both therapies. A reduced incretin effect is a well-known characteristic of patients with type 2 diabetes. Impaired release of glucagon-like peptide-1 (GLP-1) has been reported to be at least partly involved in impairment of early-phase insulin secretion after food intake and postprandial hyperglycemia and hyperlipidemia [20].

High concentrations of several antidiabetic drug classes, namely thiazolidinediones, sulphonylureas, meglitinides and morphilinoguanides have been reported to inhibit the DPP-IV enzyme, being nateglinide the strongest inhibitor. So, besides its effect as a beta-cell K-ATP channel inhibitor [6, 7], nateglinide was reported to act as a prandial insulin-releasing agent, both by inhibiting GLP-1 degradation [21, 22], and by increasing GLP-1 secretion [8] secondary to the increase of intracellular calcium.

We did not confirm these data in our study.

There was no change in blood pressure. Nateglinide, probably due to its little binding to the vascular muscle and cardiac SUR2 receptors [9], was not expected to change blood pressure levels. Overall assessment of safety demonstrated that both drugs were well tolerated, and there were no significant side effects or severe hypoglycemic episodes.

Although metformin has been confirmed as the first line option to treat diabetes, troublesome gastrointestinal intolerance sometimes precludes its use [2]. Thus, glinide remains an important adjuvant for recent onset T2D patients. When compared with rosiglitazone, nateglinide achieved similar efficacy in improving glucose control. Although rosiglitazone had a sparing effect on beta cell function and decrease FFA, triglycerides and PAI-1 levels, it was recently excluded from the marked due to an increase in cardiovascular disease. Despite leading to more insulin secretion, nateglinide did not worsen body weight or beta cell function, measured by the pro-insulin/insulin ratio. Long term studies are needed to ascertain whether it can prevent beta cell exhaustion or apoptosis.

## Conclusions

In patients with type 2 diabetes inadequately controlled by dietary therapy, nateglinide and rosiglitazone resulted in similar overall improvements in glucose control and beta cell function. In addition, nateglinide reduced total cholesterol levels and rosiglitazone reduced post-prandial FFA and triglycerides levels and some inflammatory markers. Despite different mechanisms of action nether drug changed GLP-1 levels or previous secretory dysfunction of alpha cells. Maintenance of inflammatory markers, haemostatic factors and glucagon levels can be related to the continuously worsening of cardiovascular function and glucose control observed in DM2.

## Abbreviations

CRP: Reactive protein
DM2: Type 2 diabetes mellitus
FFA: Free fat acids
GLP-1: Glucagon like peptide-1
IL-6: Interleukin 6
PAI-1: Plasminogen activator inhibitor
TGFβ: Transforming growth factor beta
TNFα: Tumor necrosis factor alfa
sICAM-1: Intercellular adhesion molecule-1
